# A Case Proving the Deleterious Effects of the Agaricus Campanulatus, Which Was Mistaken for the Agaricus Campestris, or Champignon

**Published:** 1816-12

**Authors:** G. Glen


*For the London Medical and Physical Journal.*

### *A Case proving the Deleterious Effects of the Agaricus Campanulatus, which was mistaken for the Agaricus Campestris, or Champignon.* By G. Glen, Esq.

THE subject of this paper, a poor man residing in Knightsbridge, went out on the morning of the 16th ult. into Hyde Park, with the intention of gathering some champignons. He collected a considerable number of mushrooms around a copse of young trees behind the Horse-Guard Barracks, which he believed to be of the desired kind. These he brought home, stewed, and shortly after began eating them. He had finished the whole, with the exception of about six or eight, when (about eight or ten minutes from the commencement of his meal) he was, to use his own expressions, suddenly seized with a dimness or mist before his eyes, lightness and giddiness of his head, with a general trembling and sudden loss of power,—so much so, that he nearly fell off the chair; to this succeeded, loss of recollection; he forgot where he was, and all the circumstances of his case. This temporary deprivation soon went off, and he so far rallied as to be able, though with difficulty, to get up, with the intention of coming here for assistance,—a distance of about five hundred yards: he had not proceeded more than half way when his memory again failed him; he lost the road, although previously perfectly acquainted with it, but was fortunately met by a friend, who with difficulty learned his state and brought him to me.

His countenance betrayed great anxiety; he could scarcely stand, but reeled about somewhat like a drunken man; he spoke with hesitation and reluctance; he complained of no pain except some transient twitches in his legs; he had no nausea; he suffered much from giddiness, and was greatly inclined to sleep; his pulse was slow and feeble.

Seeing the state of my patient required instant relief, I immediately gave him the following draught, viz.

℞. Zinci Sulphat. g xxv. Liqr. Antimon. tart. f. ℥i. m.

But the effects of the poison had so impaired the sensibility of the stomach, that vomiting did not take place for near twenty minutes after, although another draught of the same kind was exhibited in that time. During this interval his drowsiness increased to such a degree that he was only kept awake by obliging him to walk round the room by support; he also, at this time, complained of distressing pains in the calves of his legs.—Copious vomiting was at length produced, when a great quantity of mutilated mushroom was ejected. After the operation of the emetic, he expressed himself generally better, but still continued drowsy. In the evening I found him doing well, but gave him a cathartic, as his bowels had not been excited by the emetics.

17th Oct.—My patient is to-day nearly well, his drowsiness almost gone; he only complains of great weakness and languor. From this time he rapidly recovered his usual health.

I have procured not only some of those mushrooms which he had stewed, but also a few fresh ones from the place he obtained those he had eaten: they both agree in appearance, and have all the characteristics of the agaricus campanulatus of Linnæus; for greater certainty, as to the species, I showed them to my friend and late teacher, Mr. William Salisbury, of Sloane-street, who pronounced them to be of the above species. A similar case to the present is related in the Gentleman's Magazine for September 1815, by the same intelligent gentleman.

As we usually judge of the virulence and activity of a poison poison in proportion to the effects produced, and the time required to act, surely I am justified in the conclusion, that the agaricus campanulatus appears to be both an active and virulent poison, from the early appearance of the effects, and their rapid but regular progress throughout; also, the total absence of pain, the great inclination to sleep, and the torpidity of the stomach, evince that their effect was solely sedative. What would have been the result, had no assistance been given, is rather uncertain? this, however, is probable, that, had they not been the cause of his death, their destructive effects might have injured his health for some time to come.

*Brompton-row, Knightsbridge;*

*November* 12, 1816.

